# Ultra-confined surface phonon polaritons in molecular layers of van der Waals dielectrics

**DOI:** 10.1038/s41467-018-04168-x

**Published:** 2018-05-02

**Authors:** Alexander M. Dubrovkin, Bo Qiang, Harish N. S. Krishnamoorthy, Nikolay I. Zheludev, Qi Jie Wang

**Affiliations:** 1 Centre for Disruptive Photonic Technologies, TPI, SPMS, Nanyang Technological University, 637371 Singapore, Singapore; 2 Optoelectronics Research Centre and Centre for Photonic Metamaterials, University of Southampton, Southampton, SO17 1BJ UK; 3 OPTIMUS, Centre for OptoElectronics and Biophotonics, School of Electrical and Electronic Engineering, Nanyang Technological University, 639798 Singapore, Singapore

## Abstract

Improvements in device density in photonic circuits can only be achieved with interconnects exploiting highly confined states of light. Recently this has brought interest to highly confined plasmon and phonon polaritons. While plasmonic structures have been extensively studied, the ultimate limits of phonon polariton squeezing, in particular enabling the confinement (the ratio between the excitation and polariton wavelengths) exceeding 10^2^, is yet to be explored. Here, exploiting unique structure of 2D materials, we report for the first time that atomically thin van der Waals dielectrics (e.g., transition-metal dichalcogenides) on silicon carbide substrate demonstrate experimentally record-breaking propagating phonon polaritons confinement resulting in 190-times squeezed surface waves. The strongly dispersive confinement can be potentially tuned to greater than 10^3^ near the phonon resonance of the substrate, and it scales with number of van der Waals layers. We argue that our findings are a substantial step towards infrared ultra-compact phonon polaritonic circuits and resonators, and would stimulate further investigations on nanophotonics in non-plasmonic atomically thin interface platforms.

## Abstract (web summary)

Ultra-compact phonon polariton devices may benefit from the atomically thin nature of van der Waals materials. Here, the authors report that atomically 2D transition metal dichalcogenides on a silicon carbide substrate support a 190-fold confinement of propagating surface phonon polaritons.

## Introduction

Highly confined polaritons allow ultimate photonics integration at nanoscale landscape^1^. Bearing strong light–matter interactions, they unveil diverse classical and quantum phenomena^2,3^ and found applications in photodetection^4^, biosensing^5^, super resolution imaging^6,7^, sub-diffractional resonators^8–10^ and nanofocusing^11^. A roadmap for polaritons has been greatly enriched with a plethora of two-dimensional materials that emerged in recent years^2,12^. These structures—molecular layers stacked by van der Waals interaction—can be potentially used for nanoscale waveguiding, interferometry and subwavelength resonators, providing new functionalities and larger confinement factors compared to conventional metal-dielectric predecessors. Their applicability towards extreme squeezing of polaritonic waves has been predominantly discussed in the form of plasmon polaritons^2^, and extensively experimentally investigated in graphene^10,13,14^. More sophisticated graphene plasmon-based quasiparticles (hybrid plasmon–phonon polaritons) have been demonstrated^15–17^. At the same time, ultrahigh squeezing (reaching and exceeding 10^2^) of phonon polaritons—alternative carriers allowing low-loss nanophotonics^18^—has not been sufficiently explored. It remains a challenge as the best experimental efforts reported only several ten-times confinement (~50 utilizing boron nitride^2,19–22^ and ~70 in conventional materials^23^).

In this work, we show that two-dimensional (2D) materials allow to study ultimate limits of phonon polaritons confinement, and introduce atomically thin layers of transition-metal dichalcogenides (TMDs) on silicon carbide substrate as a new system experimentally supporting ultra-confined surface phonon polaritons (SPhPs). The physics of our system is different from commonly studied phonon polaritons in boron nitride. The latter demonstrates volume-confined hyperbolic sheet modes^19–22^, while its surface hyperbolic modes exist only at the crystal edges and have been observed only in thick samples^21^. Our SPhP system does not rely on hyperbolicity of van der Waals materials, which opens new opportunities for the confinement. Scattering-type scanning near-field optical microscopy (s-SNOM) of MoS_2_–, MoSe_2_–, WS_2_– and WSe_2_–SiC interfaces revealed mid-infrared (mid-IR) SPhPs with a wavelength of more than two orders of magnitude shorter than that of the electromagnetic radiation in free space. Exceptional uniformity of van der Waals materials allowed us to measure polariton scaling law upon varying of molybdenum disulphide layer numbers from bulk to bi-layer structures. Simultaneously, a combination of strong polar property of the substrate with large refractive indices of TMDs allows efficient spectral tunability of SPhPs by minor adjustments of the excitation wavelength. A theoretical confinement factor of the phonon polaritons may exceed three orders of magnitude at dispersion peak, which, being beyond resolution of the existing experimental tools, sketches a potential direction for nanophotonic layout miniaturization.

## Results

### Origin of ultra-confined surface polaritons

**Fig. 1:**
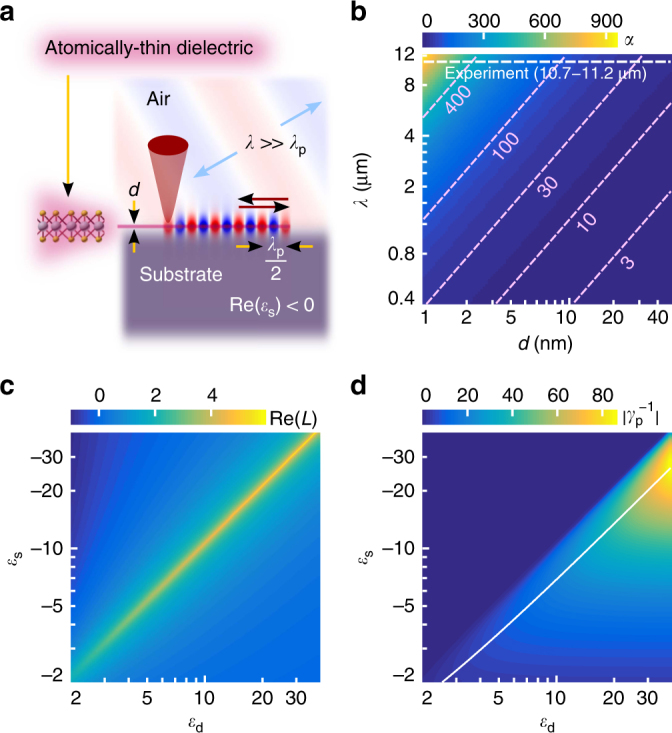
Highly confined polaritons at the three-layer interface. **a** Schematics of the experiment. **b**, **c** Tabulated plots of the factors *α* (**b**) and Re(*L*) (**c**) defined in Eq. (1). White dotted line in image (**b**) represents the span of parameters used in the experiment; pink dotted lines and corresponding numbers highlight values of the parameter *α*. **d** Tabulated plot of the figure of merit for polariton propagation losses. Solid white curve depicts a set of the figure of merit local maximums upon varying the permittivity of the substrate and the dielectric

A three-layer interface (Fig. 1a) consisting of an arbitrary low-loss substrate with negative permittivity $\left( {{\mathrm{Re}}\left( {\varepsilon _{\mathrm{s}}} \right) < 0} \right)$, a low-loss dielectric with positive permittivity $\left( {{\mathrm{Re}}\left( {\varepsilon _{\mathrm{d}}} \right) > 0} \right)$ and air (*ε*_a_=1) may support highly confined surface polaritons when the thickness of the dielectric layer is much smaller than the wavelength of light in free space. Assuming large momentum solutions $\left( {k_{\mathrm{p}} \gg k} \right)$, inherent to such polaritons, we derive a simplified expression for the complex confinement factor from an inexplicit general dispersion relation of TM-polarized surface waves (Supplementary Note 1):

1 $$\beta \simeq - \frac{\lambda }{{4{\mathrm{\pi }}d}} \times \ln \left( {\frac{{1 - \varepsilon _{\mathrm{d}}}}{{1 + \varepsilon _{\mathrm{d}}}}\frac{{\varepsilon _{\mathrm{s}} - \varepsilon _{\mathrm{d}}}}{{\varepsilon _{\mathrm{s}} + \varepsilon _{\mathrm{d}}}}} \right) \equiv - \alpha \left( {\lambda /d} \right) \times L\left( {\varepsilon _{\mathrm{s}},\varepsilon _{\mathrm{d}}} \right),$$

where $\beta = k_{\mathrm{p}}/k,$ *k*_p_ is polariton complex wavevector, *k* and *λ* are corresponding wavevector and wavelength in free space and *d* is dielectric layer thickness. As seen from Eq. (1), the confinement of polaritons, represented by Re(*β*), arises from two factors: *α* which scales proportionally to the ratio of free-space wavelength to dielectric layer thickness; and *L* resonant logarithmic term, solely depending on permittivities, dispersive in general case. Tabulated plot for $\alpha \left( {\lambda ,d} \right)$ in Fig. 1b explains extremely high confinement arising at infrared frequencies in nanometric dielectric layers on negative-permittivity substrates. Slow varying logarithmic term may further enhance the confinement, as shown in Fig. 1c for the case of low-loss materials (as an example, Im(*ε*_s_) set as 0.2, reachable for polar crystals in infrared and best plasmonic metals in visible; Im(ε_d_) set as 0, corresponding to a transparent dielectric). The figure of merit for polaritons^24,25^ propagation losses, represented by the magnitude of the ratio $\gamma _{\mathrm{p}}^{ - 1} \equiv {\mathrm{Re}}\left( {k_{\mathrm{p}}} \right)/{\mathrm{Im}}\left( {k_{\mathrm{p}}} \right) = {\mathrm{Re}}\left( L \right)/{\mathrm{Im}}\left( L \right)$, depends only on material parameters $\left( {\varepsilon _{\mathrm{s}}\left( \omega \right),\varepsilon _{\mathrm{d}}\left( \omega \right)} \right)$ and does not depend on dielectric layer thickness in contrast to relatively weekly confined modes^25,26^. As could be seen from the tabulated plot of the magnitude $|\gamma _{\mathrm{p}}^{ - 1}|$ (Fig. 1d), the strategy for low-loss polaritons lies in matching optimum conditions for the ratio of *ε*_s_ to *ε*_d_ (depicted by the white curve) and at the same time engaging low-loss materials with high permittivity. We note that the value of $\gamma _{\mathrm{p}}^{ - 1}$ is negative, indicating a negative index of the polariton mode.

### Experimental realization of ultra-confined SPhPs with van der Waals dielectrics

As a polaritonic substrate for building the three-layer interface we use undoped 6H silicon carbide. SPhPs at bare SiC–air interface appear at mid-IR frequencies, resulting from negative sign of the permittivity (Supplementary Note 2). These polaritons, however, are weakly confined (*k*_p_<2*k*^27,28^), that limits its application. Similar limitation has been recently overcome in quartz crystals by sputtering thin capping layers of germanium antimony telluride (GST), leading to strong reduction of SPhP wavelength^23^. The ultimate performance of the device, however, was limited by the film quality on several-nm thickness scale, and would also require a ‘stronger’ type of polar crystal (such as silicon carbide). In the present work, to reach an extreme confinement, we aimed to cover the SiC substrate with low-loss van der Waals dielectric layers as their atomic-level uniformity and sub-nm thickness allow a dramatic modification of the dispersion according to Eq. (1). The search for suitable candidates remains challenging, as optical constants for transparent 2D materials in mid-IR spectral range remain rather unexplored. On the other hand, a family of TMD crystals, represented by MoS_2_, MoSe_2_, WS_2_ and WSe_2_, both in bulk and monolayer form, show near-zero imaginary and large real permittivity at near-infrared (NIR) frequencies below band gap^29,30^. This trend, providing low doping to minimize free-carrier losses, places them as potentially transparent dielectrics for the part of the spectrum between NIR and intrinsic far-infrared optical phonon^31^ lines. Without limiting a general concept of our findings, we corroborate this for undoped molybdenum disulphide, which was used for major studies in this work. Dielectric permittivity, extracted from transmission measurements on suspended MoS_2_ microcrystals, shows near-flat value of 13.7 for the real part, and 0.27–0.29 dispersive imaginary part in the spectral range of polariton mapping experiments (930–897 cm^−1^). Full data are given in Supplementary Fig. 3, 4.

### Dispersion and scaling of ultra-confined SPhPs

**Fig. 2:**
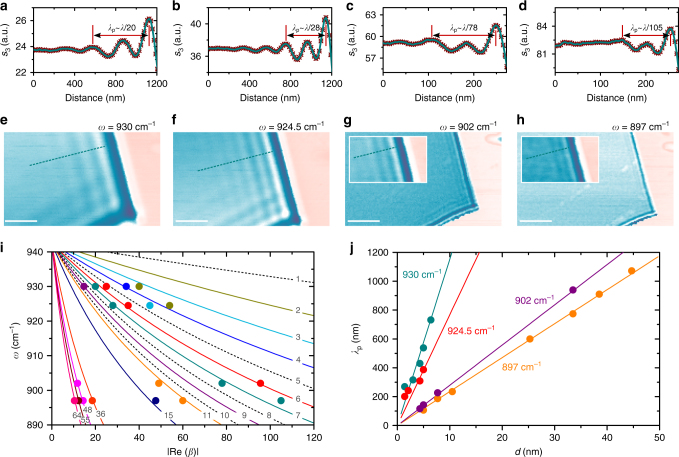
Infrared nano-imaging, dispersion and scaling of MoS_2_–SiC SPhPs. **a**–**h** Near-field images (optical amplitude *s*_3_) of 7-layer MoS_2_ on SiC (**e**–**h**) recorded at different laser excitation lines (marked as *ω*), and corresponding cross-sections (**a**–**d**) along cyan dotted lines. Cyan and pink colours refer to MoS_2_ crystal and the substrate (SiC) areas correspondingly. Cyan curves in images (**a**–**d**) serve as eye guide. Insets in images (**g**,** h**) show zoomed in fringes on the top right hand side part of MoS_2_–SiC boundary. Scale bars are 500 nm. **i** Calculated (solid/dotted curves) and experimental (circles) dispersion for different number of MoS_2_ layers (labelled on each of the curves). Data corresponding to the same number of layers are marked with the same colour. **j** Calculated and experimental scaling of the SPhPs wavelength with MoS_2_ thickness. Data corresponding to the same laser line are marked with the same colour

Real-space sub-diffractional imaging of highly confined polaritons requires an experimental tool capable to overcome large momentum mismatch between the surface wave and free-space radiation. s-SNOM^32^ has demonstrated exceptional opportunities for this application, establishing polariton interferometry technique for mapping propagating surface waves at nanoscale^13,14,19,33^. Here, a sharp metallic tip works as deeply subwavelength antenna for simultaneous launching and detection of polaritons electric field (as sketched in Fig. 1a). The tip-launched wave travels to an edge of a crystal, reflects back and forms a standing polariton pattern, which is mapped by the tip upon scanning the sample. Double period of the pattern corresponds to a polariton wavelength, *λ*_p_. At the same time s-SNOM allows direct topographic identification of TMD layers, as it is based on a high resolution atomic-force microscope (see Methods for more experimental details).

s-SNOM infrared nano-imaging of the TMD crystals transferred on silicon carbide revealed extremely confined propagating phonon polaritons predicted by the theory. While SPhP wavelength was found to be easily tunable by the excitation frequency, it also dramatically scales with the thickness of the crystal. We observe a good agreement between the theoretical dispersion, governed by Eq. (1), and s-SNOM data obtained for MoS_2_–SiC interface down to four van der Waals layers of the 2D material (Fig. 2i). Solid and dotted curves are calculated for discrete thickness of the crystal, given in numbers of MoS_2_ layers (a single-layer thickness is set as 0.7 nm^34^). s-SNOM images of 7-layer MoS_2_ (~5 nm thick) and corresponding near-field signal cross-sections are shown in Fig. 2a–h (topography is given in Supplementary Fig. 5). For each of the images, recorded at 930, 924.5, 902 and 897 cm^−1^ excitation laser lines, we can clearly see from three to six SPhP interference fringes. Assuming that the fringe period corresponds to half wavelength of the polariton (*λ*_p_/2-model^13,14,33^), we obtained a good agreement between theoretical and experimental values for the confinement factor Re(*β*). A minor (~3.6%) adjustment of the excitation wavelength between frequencies of 930 and 897 cm^−1^ leads to a considerable large (~500%) tuning of the *λ*_p_ value. At the same time, SPhPs are featured by confinement factors exceeding two orders of magnitude (e.g. 105, as in Fig. 1d, h). We observe similar ultra-confined tunable polaritons in other van der Waals materials of the TMD family (MoSe_2_, WS_2_ and WSe_2_), and we also report SPhPs in deeply sub-diffractional finite TMD structures. Several examples are given in Supplementary Fig. 6 and Supplementary Note 3. In addition, we also estimated the figure of merit for MoS_2_ SPhP propagation loss (Supplementary Note 4). As an example, $\gamma _{\mathrm{p}}^{ - 1}$ reaches a magnitude of ~13 at 897 cm^−1^ (as extracted from the best quality data).

At a fixed frequency the dispersive parameter $L\left( {\varepsilon _{\mathrm{s}},\varepsilon _{\mathrm{d}}} \right)$ in Eq. (1) becomes a constant, and SPhP confinement factor is proportional to the ratio *λ*/*d*. This fundamental scaling law can also be rewritten in terms of SPhP wavelength (namely: $\lambda _{\mathrm{p}} \simeq 4{\mathrm{\pi }}d/{\mathrm{Re}}\left( L \right)$), predicting a proportional shrinking of *λ*_p_ upon reducing of the dielectric thickness. The experimental data, plotted in *λ*_p_(*d*) representation (Fig. 2j), well support this scaling law down to four layers of molybdenum disulphide. As an example, we observed the polariton wavelength as short as 106 nm in 7-layer MoS_2_, 599 nm in 36 layers, and 1072 nm in 64 layers (at 897 cm^−1^ excitation laser line).

**Fig. 3:**
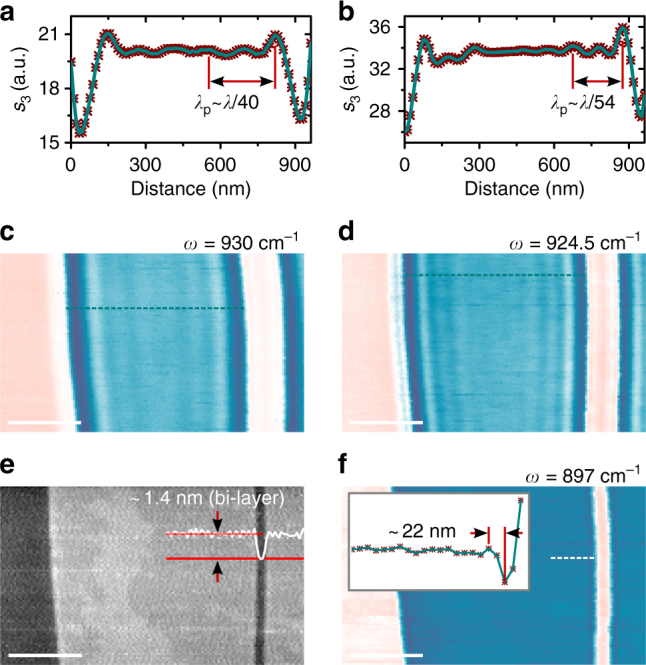
Highly confined SPhPs on bi-layer MoS_2_–SiC structure. **a**–**d** Near-field images (**c**,** d**) and corresponding cross-sections (**a**,** b**) along cyan dotted lines, recorded at *ω* = 930 and 924.5 cm^−1^. **e** AFM topography of the sample. Inserted white profile shows the height variation along the bottom red line. **f** Near-field image at *ω* = 897 cm^−1^. Inset in image (**f**) shows cross-section of the near-field signal along the dotted white line. Scale bars are 400 nm

Remarkably, MoS_2_ crystals are capable to highly confine silicon carbide phonon polaritons even when their thickness is as small as 1.4 nm (~*λ*/8000, which corresponds to a bi-layer structure) as demonstrated in Fig. 3. This opens new opportunities in SPhP-based layout miniaturization, interconnecting atomically thin structures with ultra-confined states of light. Interestingly, we found that the scaling of the polariton wavelength in tri- and bi-layer MoS_2_ non-negligibly deviates from the law observed in thicker structures. For example, polaritons at *ω*=924.5 cm^−1^ exhibit nearly 50% smaller confinement factor (54 instead of 101) than the value given by the proportional scaling dependency governed by Eq. (1). Such deviation indicates a change in MoS_2_ permittivity with thickness in the SPhP experiment. Indeed, it is known that optical and electronic properties of molybdenum disulphide gradually deviate from the bulk response when the thickness of the material is reduced below 4–6 van der Waals layers^34,35^. Furthermore, the effects of a possible thin (~1 nm) native oxide layer^36^ on the surface of silicon carbide may contribute to the deviation of experimental and theoretical data at low thickness of MoS_2_ crystal.

We note that the theoretical confinement factor, Re(*β*), calculated for MoS_2_–SiC interfaces according to Eq. (1), exceeds three orders of magnitude (the plot of confinement is given in Supplementary Fig. 9). This is interesting as it potentially allows extremely confined polaritonic systems. An experimental corroboration of such large values for propagating waves is yet limited by the resolution of existing experimental tools, and has not been reported in literature to the best of our knowledge. In s-SNOM this limit is further challenged by polariton propagation geometrical losses and launching/reflection efficiency^23,24^. For the highest confinement factors reported in this work, the measurements are performed close to the tip size limited regime. As an example, in Fig. 2h the experiment is carried out with comparable values of s-SNOM tip size and *λ*_p_/2 (both ~50 nm), while the ultimate resolution of the technique is in the order of 10 nm^37^. For experimental confinement points in Fig. 2i, j we extracted the polariton wavelengths from data where at least three interferometric fringe peaks (which corresponds to one *λ*_p_) are visible, explicitly demonstrating periodicity of the waves. This is not the only method used in literature. For example, the highest reported^23^ SPhP confinement factors (~50–70) on quartz/GST interface have been extracted from the near-field signal cross-sections which contained only one visible single-fringe feature (experimental data can be found in the Supplementary Information of ref. ^23^). In our case, s-SNOM images, where theory predicts more than 100 times confinement (e.g., Fig. 3f and Supplementary Fig. 5c), also contain frequency-dependent single-fringe features near the edge of MoS_2_ crystal. Detailed analysis (Supplementary Note 5) shows extremely short-wavelength polaritons in bi-layer MoS_2_ at *ω*=897 cm^−1^, featured by the confinement factor ~190. This corresponds to ~22 nm distance between the first minimum of the near-field signal inside MoS_2_ crystal and the neighbouring maximum inside the crystal (inset in Fig. 3f).

## Discussion

In summary, we have demonstrated for the first time that interfaces of atomically thin van der Waals dielectrics on polar substrate support ultra-confined surface phonon polaritons. While the observed extreme confinement is apparently beneficial for miniaturization of photonic layouts, the polaritons are also highly dispersive which efficiently provides a spectral tunability of the device optical response. As TMDs can be grown in large scales with exceptional crystal quality (important for unperturbed polaritons excitation), we anticipate a technological advantage of our approach in real-life applications. The revealed SPhP wavelength scaling with the number of van der Waals layers gives insight into the fundamental limits of atomically thin non-plasmonic systems supporting strong light–matter interactions. We foresee a potential application of the introduced platform in the development of building blocks for ultra-compact nanophotonic circuits. Based on highly dispersive behaviour of MoS_2_–SiC interfaces, described in detail above, we predict a narrow resonance linewidth of SPhP nanoresonators and correspondingly high-quality factors which are important for practical applications.

## Methods

### Near-field microscopy

Our setup is based on a commercial s-SNOM (Neaspec GmbH), which allows single laser-line mid-IR nano-imaging simultaneously with the sample topography mapping. The s-SNOM incorporates tapping-mode atomic-force microscope (AFM) performing measurements with Arrow-NCPt (Nanoworld) metal-coated Si tips, oscillating at frequency of ~270–285 kHz with 70–90 nm amplitude. Temperature-tunable CO_2_ laser (Access Laser) radiation is focused on the sample in oblique configuration. Pseudo-heterodyne interferometric detection at nonlinear harmonics of the tapping frequency^38^ (third in this work) allows us to measure amplitude (*s*_3_) and phase of the tip-scattered signal, which represent near-field distribution at the sample. Cross-sections of the optical signal (taken always along the fringe evolution direction) were obtained by averaging several neighbouring lines in order to increase signal-to-noise ratio.

### Sample fabrication

Undoped TMD materials (2D Semiconductors Inc.) were mechanically exfoliated onto undoped (semi-insulating) 6H silicon carbide wafers (PAM-XIAMEN Ltd). The thickness of the resulting van der Waals TMD crystals was identified by the AFM incorporated in s-SNOM setup.

### Molybdenum disulphide permittivity

The dielectric function of MoS_2_ was extracted from transmission spectrum by Swanepoel’s method^39^. A thin free-standing MoS_2_ flake was exfoliated from the bulk crystal. The transmission spectrum of the flake was measured with Bruker VERTEX 80V FTIR spectrometer. Similar to the procedures described in the literature^40^, a real part of the refractive index (*n*) was calculated from envelop of peaks and valleys of the spectrum. A thickness (*t*) was determined from the interference pattern and calculated (*n*). An imaginary part of the refractive index was calculated from the spectrum envelop and *t*.

### Data availability

The data from this paper can be obtained from the University of Southampton ePrints research repository, 10.5258/SOTON/D0470.

## Electronic supplementary material

Supplementary Information
