# Supplementary material for: Ultra-confined surface phonon polaritons in molecular layers of van der Waals dielectrics
Source: Nat Commun. 2018 May 2;9:1762. doi: 10.1038/s41467-018-04168-x (PMC5932077; doi:10.1038/s41467-018-04168-x)
Supplement: Supplementary file 1 — Supplementary Information [file 41467_2018_4168_MOESM1_ESM.pdf]

Supplementary Information

Ultra-confined surface phonon polaritons in molecular layers of van der Waals dielectrics

Alexander M. Dubrovkin[1*], *Bo Qiang*[1], *Harish N. S. Krishnamoorthy*[1], *Nikolay I. Zheludev*[1,2*], *Qi Jie Wang*[1,3*]

[1]Centre for Disruptive Photonic Technologies, TPI, SPMS, Nanyang Technological University, 637371 Singapore
[2]Optoelectronics Research Centre and Centre for Photonic Metamaterials, University of Southampton, SO17 1BJ, UK
[3]OPTIMUS, Centre for OptoElectronics and Biophotonics, School of Electrical and Electronic Engineering, Nanyang Technological University, 639798 Singapore

*Correspondence to: dubrovkin@ntu.edu.sg, niz@orc.soton.ac.uk, qjwang@ntu.edu.sg

Supplementary Note 1. Derivation of the polariton confinement factor expression.

A dispersion of TM-polarized surface wave at the three-layer interface is given by the following inexplicit relation[1]:

$$\mathrm{e}^{-2k_1 d} = \frac{k_1/\varepsilon_1 + k_2/\varepsilon_2}{k_1/\varepsilon_1 - k_2/\varepsilon_2} \times \frac{k_1/\varepsilon_1 + k_3/\varepsilon_3}{k_1/\varepsilon_1 - k_3/\varepsilon_3} \quad (1)$$

where $k_\mathrm{i}^2 = k_\mathrm{p}^2 - k^2\varepsilon_\mathrm{i}$ (2)

$\mathrm{i} = 1,2,3$ represents the layer number; k_p and k polariton complex wavevector and free space wavevector correspondingly, d – intermediate layer ($\mathrm{i} = 1$) thickness. Assuming large momentum solutions ($k_\mathrm{p} \gg k|\sqrt{\varepsilon_\mathrm{i}}|$), Supplementary Equation (2) can be reduced to $|k_\mathrm{i}| \simeq |k_\mathrm{p}|$, which allows to simplify Supplementary Equation (1) to the explicit representation:

$$\beta \simeq -\frac{\lambda}{4\pi d} \times \ln\left(\frac{1-\varepsilon_\mathrm{d}}{1+\varepsilon_\mathrm{d}}\,\frac{\varepsilon_\mathrm{s}-\varepsilon_\mathrm{d}}{\varepsilon_\mathrm{s}+\varepsilon_\mathrm{d}}\right) \equiv -\alpha(\lambda/d) \times L(\varepsilon_\mathrm{s}, \varepsilon_\mathrm{d}) \quad (3)$$

where we introduced dimensionless complex confinement factor as the ratio between polariton and free space wavevectors ($\beta = k_\mathrm{p}/k$), and set $\varepsilon_1 = \varepsilon_\mathrm{d}$, $\varepsilon_2 = \varepsilon_\mathrm{s}$ and $\varepsilon_3 = \varepsilon_\mathrm{air} = 1$; λ depicts the wavelength in free space; the minus sign before $\alpha \times L$ term originates from the choice of $k_\mathrm{i} \simeq -k_\mathrm{p}$ representing physically propagating mode. We also confirmed, that for confinement factors ($|\mathrm{Re}(\beta)| > 10$) and the two-dimensional material (MoS_2) discussed in the main text, direct numerical solutions of Supplementary Equation (1) are almost indistinguishable from results given by simplified Supplementary Equation (3); examples of the approximated versus exact confinement plots are shown in Supplementary Fig. 1a. The figure of merit for polaritons propagation is more sensitive to the approximation procedure, however the difference in exact and approximated solutions is negligible even for several tens layers of MoS_2 (Supplementary Fig. 1b).

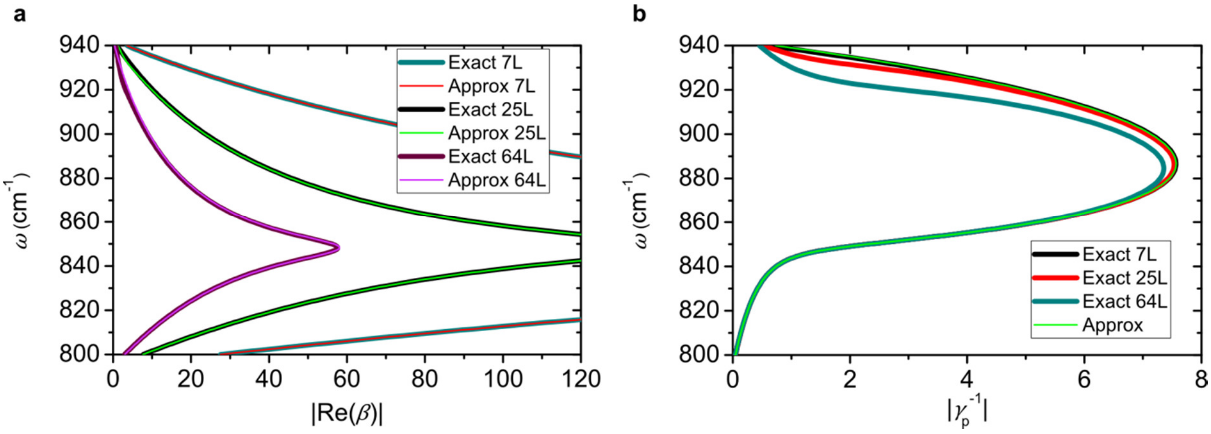

Supplementary Figure 1 | Comparison of solutions of the exact Supplementary Equation (1) and its large-momentum approximation Supplementary Equation (3). a,**b**, plots of the confinement and the figure of merit for polaritons propagation, correspondingly. Insets show number of layers used for calculations.

Supplementary Note 2. Silicon carbide permittivity.

The dielectric function of 6H–SiC is calculated using a single harmonic oscillator model: $\varepsilon_{\mathrm{SiC}} = 6.49 + \frac{3.23}{1-\left(\frac{\omega}{\omega_{\mathrm{TO}}}\right)^2 - \mathrm{i}\frac{\gamma\omega}{\omega_{\mathrm{TO}}^2}}$, with ω_{TO} = 788 cm^{-1} and γ = 6.8 cm^{-1}. These parameters are taken from literature[2] and well fit control reflection spectra of our SiC substrates in mid-IR.

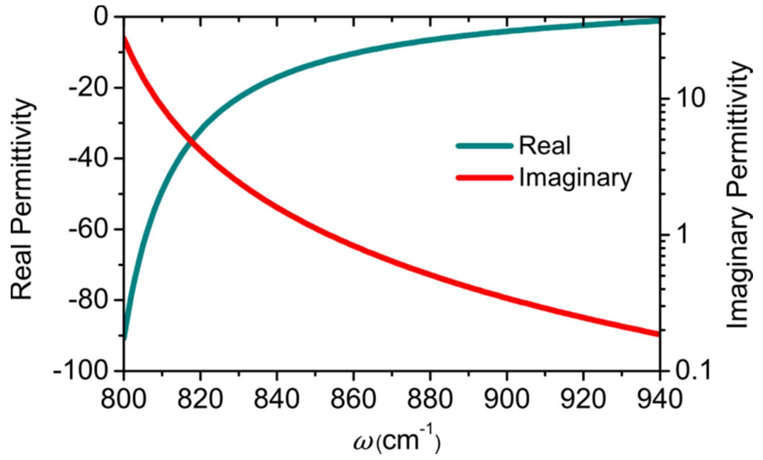

Supplementary Figure 2. Real and imaginary permittivity of SiC substrate used for calculations.

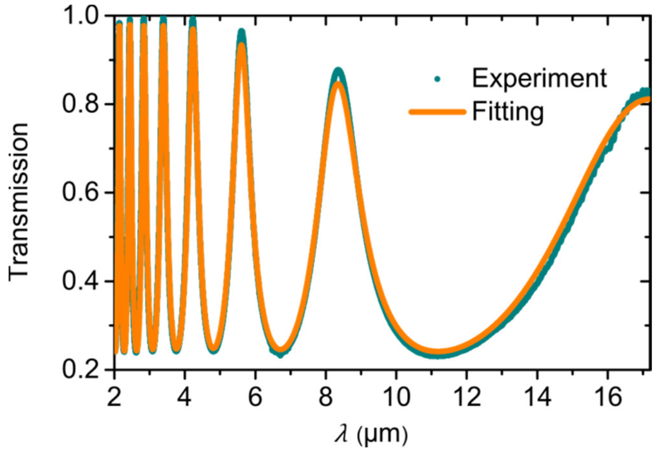

Supplementary Figure 3. Transmission of suspended MoS_2 microcrystal: experimental data and fitting.

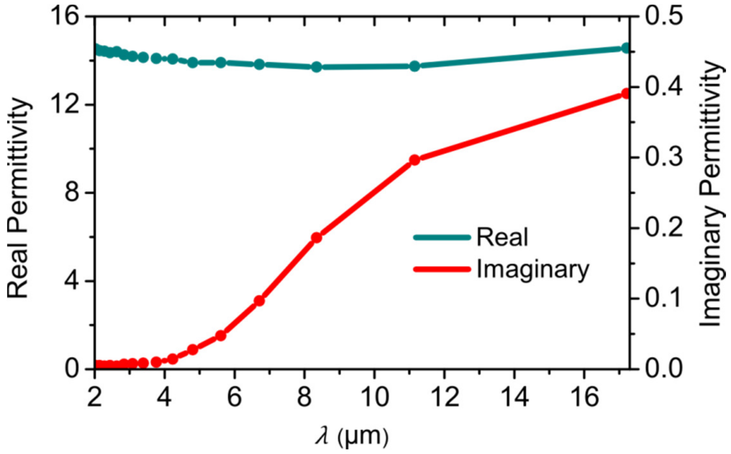

Supplementary Figure 4. Real and imaginary permittivity of MoS_2 providing the fit of the transmission data plotted in Supplementary Fig. 3.

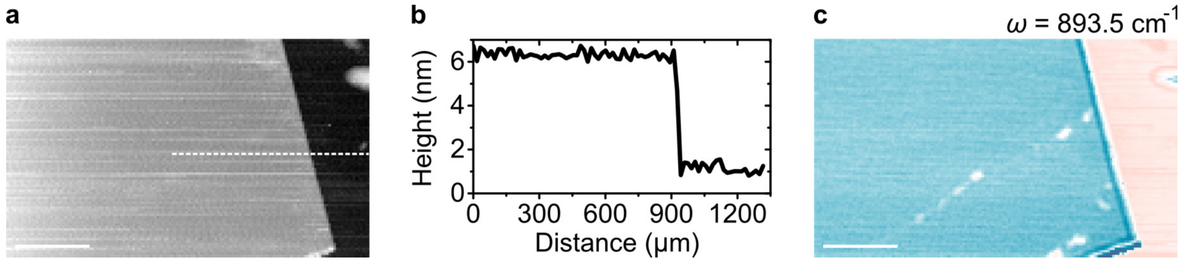

Supplementary Figure 5 | Additional data for the MoS_2 crystal, presented in Fig. 2 of the article main text. a, AFM topography of the MoS_2 crystal. **b**, Height profile along white dotted line in image **a**. **c**, s-SNOM optical image (s_3 signal), recorded at $\omega = 893.5$ cm^{-1}. Scale bars are 500 nm.

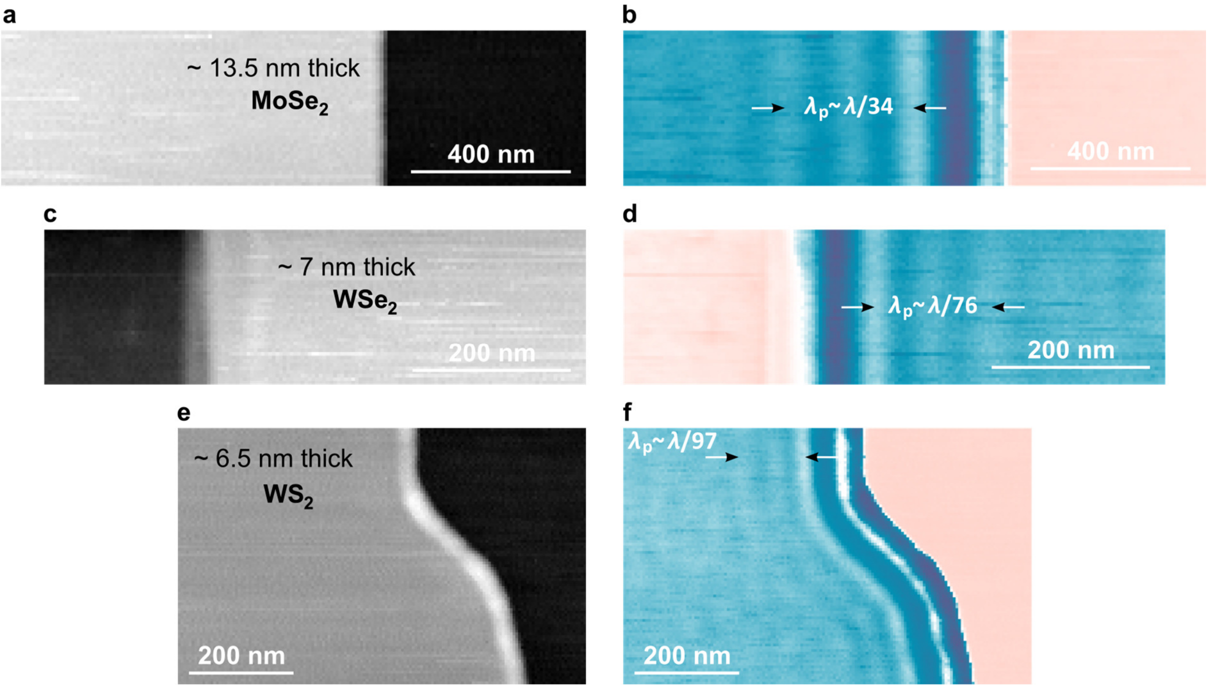

Supplementary Figure 6 | Ultra-confined phonon polaritons in $MoSe_2$, WSe_2 and WS_2 on silicon carbide substrate. a,**c**,**e**, AFM topography of the crystals. **b**,**d**,**f**, s-SNOM optical images, recorded at ω = 897 cm^{-1}, corresponding to panels **a**,**c**,**e**.

Supplementary Note 3. Highly-confined SPhPs in finite sub-diffractional TMD structures on SiC.

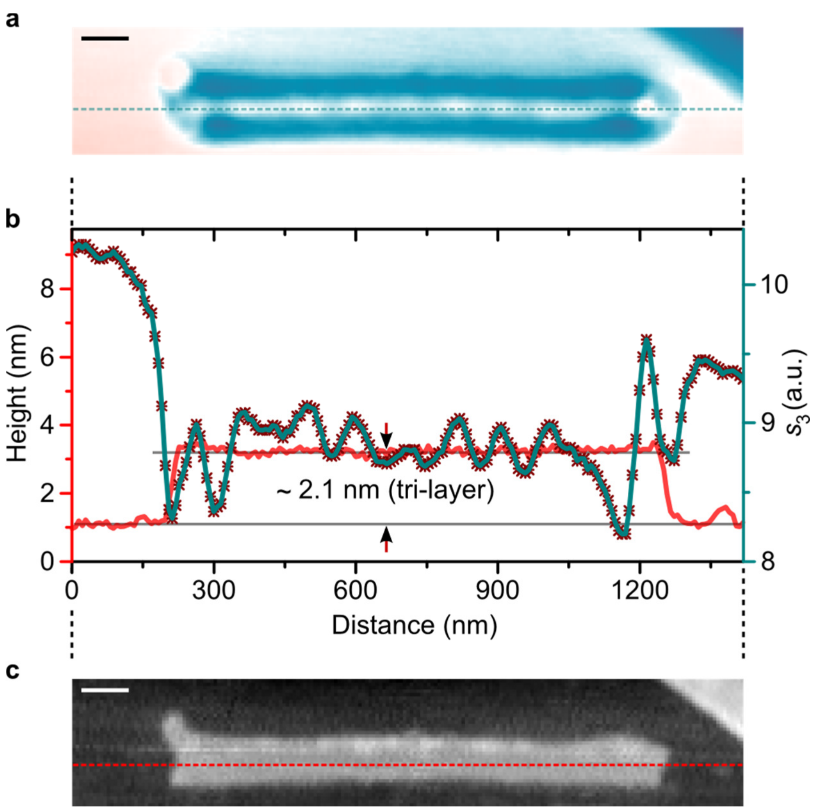

Supplementary Figure 7. SPhP mode in tri-layer MoS_2 nanoribbon on SiC. a-c, Near-field optical image (**a**), topography (**c**) and corresponding central cross-sections (**b**) along dotted cyan and red lines. Cyan colour curve in image (**b**) represents the optical signal, aligned with the topography profile (red colour). Scale bars are 100 nm. Excitation laser line: $\omega = 930$ cm^{-1}.

In addition, we report highly confined SPhPs modes in deeply sub-diffractional TMD crystals on silicon carbide substrate. As an example, a tri-layer nanoribbon with a width of approximately 100 nm (which is less than $\lambda/100$), a length of ~1 μm ($<\lambda/10$) and MoS_2 thickness of 2.1 nm is shown in Supplementary Fig. 7. The structure was fabricated in the process of the layered material exfoliation. The mode inside the ribbon is characterized by deeply subwavelength confinement, and is featured by a period ~100 nm. We believe these experimental data may be of interest for potential future applications such as nanoscale resonators or waveguides.

Supplementary Note 4. Estimate for MoS_2–SiC phonon polaritons propagation loss.

The measured fringes are fitted into a model developed in literature[3]:

$$\frac{Ae^{2ik_{\mathrm{p}}x}}{\sqrt{x}} + \frac{Be^{ik_{\mathrm{p}}x}}{x^a} + C_1 + C_2 e^{-\gamma x} \tag{4}$$

where fitting parameters A, B, k_{p} are complex numbers and a, C_1, C_2, γ – real; x – distance from s-SNOM tip to MoS_2 edge.

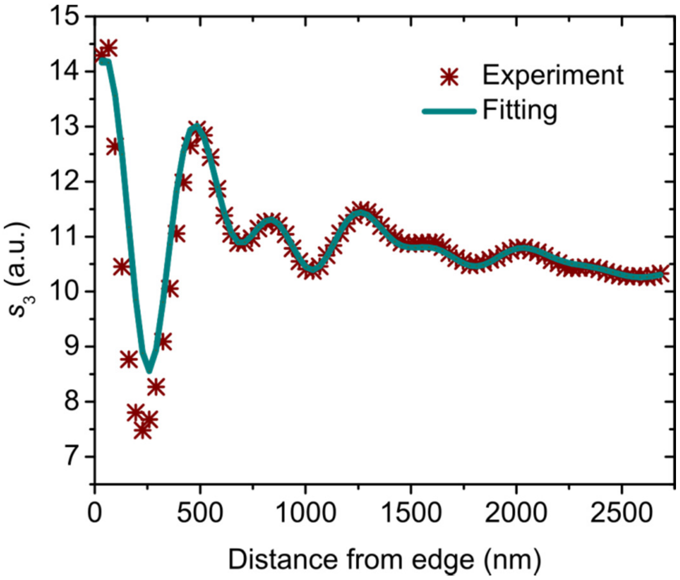

Supplementary Figure 8. Experimental data (red dots) and corresponding fitting (cyan curve) performed for the optical fringe cross-section, recorded near the edge of 48-layer MoS_2 crystal at ω = 897 cm^{-1}. Corresponding parameters of the fitting: $k_{\mathrm{p}} = (8.12 \times 10^6 + 6.30 \times 10^5 \mathrm{i})\ \mathrm{m}^{-1}$, $A = -3.55 + 43.10\mathrm{i}$, $B = 2.86 - 27.80\mathrm{i}$, $C_1 = -31.51$, $a = 0.52$, $\gamma = 1.44 \times 10^4\ \mathrm{m}^{-1}$, $C_2 = 20$.

The first term represents tip-launched surface waves. These waves travel a total distance of $2x$ (from tip to edge, and back to tip) and experience a geometry decay of $\sqrt{x}$ when the wavefront expands circularly. The second term represents all possible channels of launching/detection of the surface wave that travels only a single edge-tip distance x. The x^a term in the denominator accounts for the geometrical decay of such waves and left as a fitting parameter. The third term represents a background signal, and the fourth – possible fast SPhP modes coexisting on SiC (later can be approximated by an exponential decaying function since the wavelength of the fast modes is much larger than the range of ultra-confined (slow) modes mapping). The first period of the fringe is excluded from the fitting optimization as it would overcomplicate the model (it is known that complex tip-edge coupling near an edge discontinuity cannot be modelled straightforwardly[3] without providing additional assumptions). Example of the modelling is shown in Supplementary Fig. 8; corresponding estimate for the SPhPs propagation loss figure of merit is ~13. In addition, we note that the weight of edge-launched versus tip-launched contribution may depend on particular geometry/shape of the crystal edge termination, and the tip.

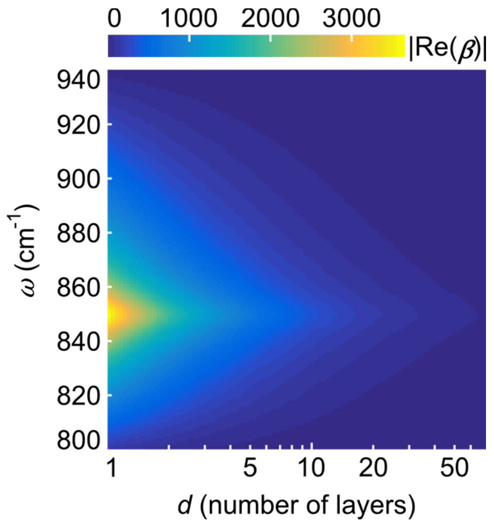

Supplementary Figure 9. SPhPs confinement factor, |Re(β)|, calculated for MoS_2–SiC interfaces according to the equation (1) of the article main text as a function of the two-dimensional material thickness (plotted in number of van der Waals layers) and the excitation frequency.

Supplementary Note 5. Analysis of the phonon polariton wavelength based on single-fringe features near MoS_2 crystal edge.

It is known[3] that complex tip-edge coupling near an edge discontinuity may result in that the first fringe (nearest to the edge) is related to the polariton wavelength (or confinement) in more sophisticated way compared to all sequent fringes further inside the crystal (later fringes can be well modelled by Supplementary Equation (4), and provide most accurate information on λ_p). From the other hand, as the first fringe scales in size with the laser excitation frequency, it may straightforwardly provide an estimate for λ_p (or $\mathrm{Re}(\beta)$) if: imaging is spectroscopically carried out at several frequencies, and the wavelength value is priory-known accurately at least at one of these frequencies.

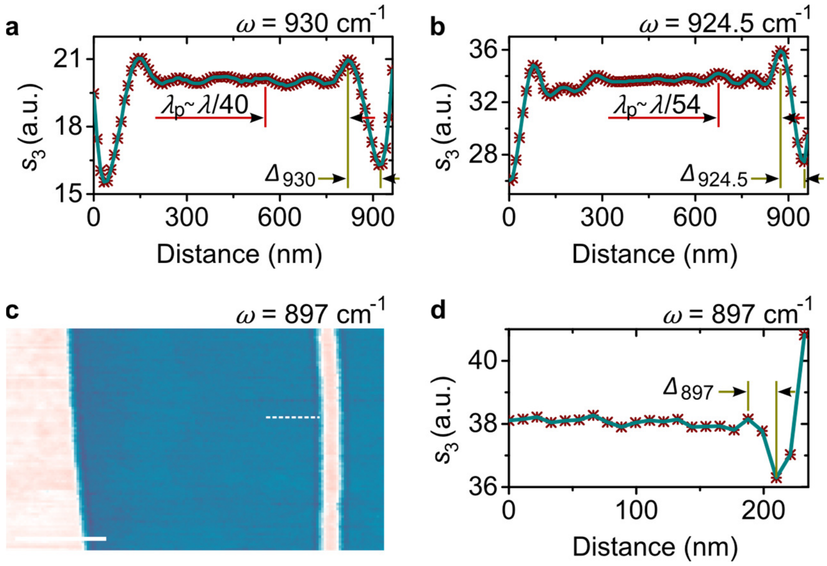

Supplementary Figure 10 | Additional analysis of bi-layer MoS_2–SiC data. **a,b,c**, Re-plot of the main text Fig. 3a,b,f with highlighted single-fringe features near MoS_2 crystal edge (olive-colour marks). Scale bar (image **c**) is 400 nm. **d**, Cross-section along the white dotted line in image **c**.

We analyse the distance (Δ) between first minimum of the scattering signal inside MoS_2 crystal edge and the neighbouring maximum further inside the crystal (Supplementary Fig. 10; Δ represents a semi-width of the first interferometric fringe, clearly defined on the experimental data). Between frequencies of 930, 924.5 and 897 cm^{-1} Δ scales as 104, 78 and 22.1 nm correspondingly. The values of $\mathrm{Re}(\beta)$ at 930 and 924.5 cm^{-1} are priory known (40 and 54, correspondingly) from the analysis of inner fringes depicted with red-colour arrows in Supplementary Fig. 10a,b. Assuming that Δ linearly depends on λ_p we observe a good agreement between priory-known values and re-calculated ones using scaling of the parameter delta (i.e. $|\mathrm{Re}(\beta)|_{924.5} = |\mathrm{Re}(\beta)|_{930}\,\frac{\Delta_{930}}{\Delta_{924.5}} = 53.3 \approx 54$), which demonstrates suitability of this method (in addition we confirmed that the other data, e.g. for 7-layer MoS_2, also follow this trend). Applying the same calculation technique to the bi-layer MoS_2 data at 897 cm^{-1} we obtain an estimate for the confinement factor, which is $|\mathrm{Re}(\beta)|\sim190$.

Supplementary References

1. Maier, S. A. *Plasmonics: Fundamentals and Applications* (Springer Science + Business Media, New York, 2007).
2. Renger, J., Grafström, S., Eng, L. M., & Hillenbrand, R. Resonant light scattering by near-field-induced phonon polaritons. *Phys. Rev. B* **71**, 075410 (2005).
3. Woessner, A. *et al.* Highly confined low-loss plasmons in graphene–boron nitride heterostructures. *Nature Mater.* **14**, 421–425 (2015).
